# RhodaMal Dyes: Novel Red Emissive Fluorophores as Probes for High Contrast Live‐Cell Imaging

**DOI:** 10.1002/chem.71410

**Published:** 2026-07-18

**Authors:** A. Mavroskoufis, P. Aßmann, L. M. Filla, I. Bilici, E. Quaas, M. Maglione, S. Sigrist, R. Haag, K. Licha

**Affiliations:** ^1^ Institute of Chemistry and Biochemistry Freie Universität Berlin Berlin Germany; ^2^ SupraFAB Freie Universität Berlin Berlin Germany; ^3^ Institute For Biology/Genetics Freie Universität Berlin Berlin Germany

**Keywords:** dyes/pigments, fluorescence spectroscopy, fluorescent probes, imaging agents

## Abstract

Red and near‐infrared emitting dyes are of high interest in fluorescence microscopy. Herein, we report two new red emissive dyes, namely RhodaMal 620 and RhodaMal 638 by incorporation of malononitrile, a highly electron‐withdrawing and π‐extending moiety into the traditional rhodol framework. The dyes have their absorption and emission maxima at 620/638 and 664/708 nm in aqueous solution. By increasing the viscosity of the medium to mimic the microenvironment of crowded and viscous subcellular compartments, we showed a drastic 20‐fold increase in the fluorescence signal. Conjugating the dyes to triphenylphosphonium cation (TPP^+^) enabled efficient mitochondrial targeting. RhodaMal 620 was further used in live cell STED microscopy with a 775 nm depletion laser, enabling super resolution with raw full‐width at half maximum values down to 77 nm.

## Graphical abstract

Incorporation of malononitrile into the traditional rhodol framework furnishes RhodaMal (Rhoda = Rhodamine, Mal = Malononitrile;) dyes, a new class of red emissive push–pull chromophores. Conjugating them to triphenylphosphonium cation enables live cell super‐resolution STED microscopy with a 775 nm depletion laser.

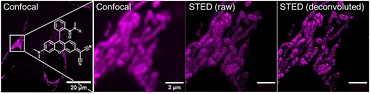

## 1 Introduction

Fluorescence microscopy has become an indispensable tool in life sciences, enabling the visualization of dynamic biological processes at subcellular resolution with high sensitivity and specificity. Advances in super‐resolution techniques such as stimulated emission depletion (STED) microscopy have further pushed the boundaries of spatial resolution beyond the diffraction limit, requiring fluorophores with tailored properties regarding their emission profiles and photostability [1, 2, 3]. Among these, fluorescent reporters that absorb and emit in the red to near‐infrared spectral window (600–900 nm) are particularly valuable [4, 5, 6, 7]. This spectral region minimizes autofluorescence, reduces phototoxicity, and allows deeper tissue penetration, making such probes highly desirable for live‐cell and deep‐tissue imaging applications [8, 9]. While cyanines and xanthene derivatives have dominated this space, there is a persistent challenge for bright, photostable, and red‐shifted dyes that meet the demands of live‐cell and super‐resolution applications [10].

Rhodols, hybrid fluorophores combining features of rhodamines and fluoresceins, have been known for over a century, but have received a renewed attention in recent decades [11]. Their electronic asymmetry, which may result in desirable properties such as large stokes shifts, makes them an attractive alternative to their parent analogues, which have been extensively used in live cell STED microscopy [12, 13]. Native rhodol derivatives, however, typically absorb and emit in the green to orange spectral region [14]. Through recent years, several strategies have been employed to red‐shift rhodol‐based fluorophores, including heteroatom substitution and extension of π‐conjugation (Figure 1) [15, 16, 17, 18, 19].

**FIGURE 1:**
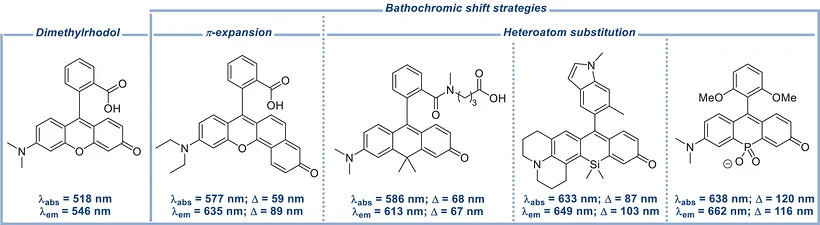
Traditional strategies for bathochromic shifts of rhodol‐based dyes.

In this context, we present a new class of red emissive push–pull rhodol analogues achieved by substituting the traditional ketone moiety with malononitrile, a highly electron‐withdrawing and conjugation‐extending group, furnishing RhodaMal dyes. The strong electron‐withdrawing nature significantly decreases the frontier molecular orbital (FMO) energy gap, while its two cyano groups enhance π‐conjugation, resulting in a substantial bathochromic shift [20]. Conjugating them to triphenylphosphonium (TPP^+^) cation provided a novel type of imaging probe for high contrast and super resolution detection in living HeLa cells.

## 2 Results and Discussion

To assess the impact of introducing the malononitrile moiety into the rhodol framework, we initially performed density functional theory (DFT) calculations at the B3LYP/6‐31+G(d,p) level with optimized geometries of both Dimethylrhodol **1** (Figure 2, left; Table S1) and the substituted malononitrile dye **2** (Figure 2, right, Table S2) [21]. The results show that the energies of the highest occupied molecular orbitals (HOMO) of the two molecules are nearly identical. However, the lowest unoccupied molecular orbital (LUMO) of the envisioned structure **2** is significantly stabilized, leading to a notable reduction in the HOMO–LUMO energy gap by 0.658 eV. This reduction should induce a substantial bathochromic shift, as the absorption and emission profiles of fluorophores are directly correlated to their HOMO–LUMO gaps, with narrower gaps corresponding to longer wavelength profiles.

**FIGURE 2:**
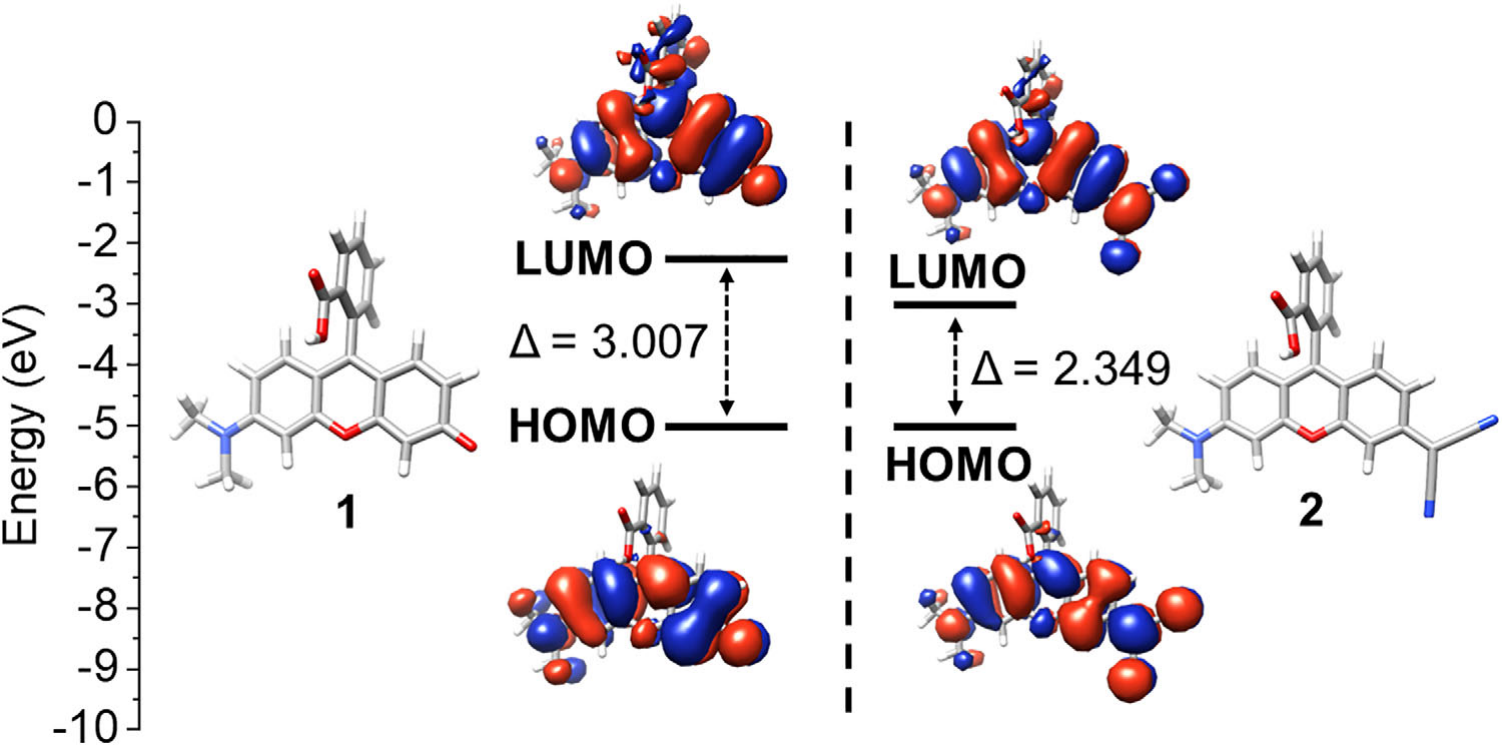
Energy levels and respective FMO's of optimized geometries for dimethylrhodol (left) and malononitrile substituted rhodol (right) calculated by DFT at the B3LYP/6‐31+G(d,p) level with Orca.

Based on these calculated results, we proceeded to synthesize the malononitrile‐substituted rhodol analogues. In initial attempts, we envisaged direct condensation of malononitrile with **1** under various conditions (piperidine, DiPEA, AcOH) or using the established titanetetrachloride/pyridine system [22, 23, 24]. However, all methods utilized proved ineffective. The stubborn reactivity may be attributed to the electron‐donating and phenolic character of the rhodol core, impeding electrophilic condensations. Shifting our strategy, we turned our attention to a report by Gao and coworkers of palladium‐catalyzed reaction of activated methylenes [25]. The required aryl bromide **3** was readily available from condensing literature known keto acid **4** with *m*‐bromophenol in good yield (Figure 3A) [26]. Followings Gao's protocol, employing PdCl_2_, PPh_3_, and NaH, the target compound **2** was isolated in modest yield. To inhibit the known open‐closed equilibrium behavior characteristic for the parent rhodols and rhodamines (Figure 3), which are known to undergo with primary and secondary amides covalently linked to the ortho position of the pendant phenyl ring, the carboxylic acid was converted to the tertiary amide using *N*‐methyl ester **5**, which was subsequently saponified, yielding final RhodaMal 620 **6** dye.

**FIGURE 3:**
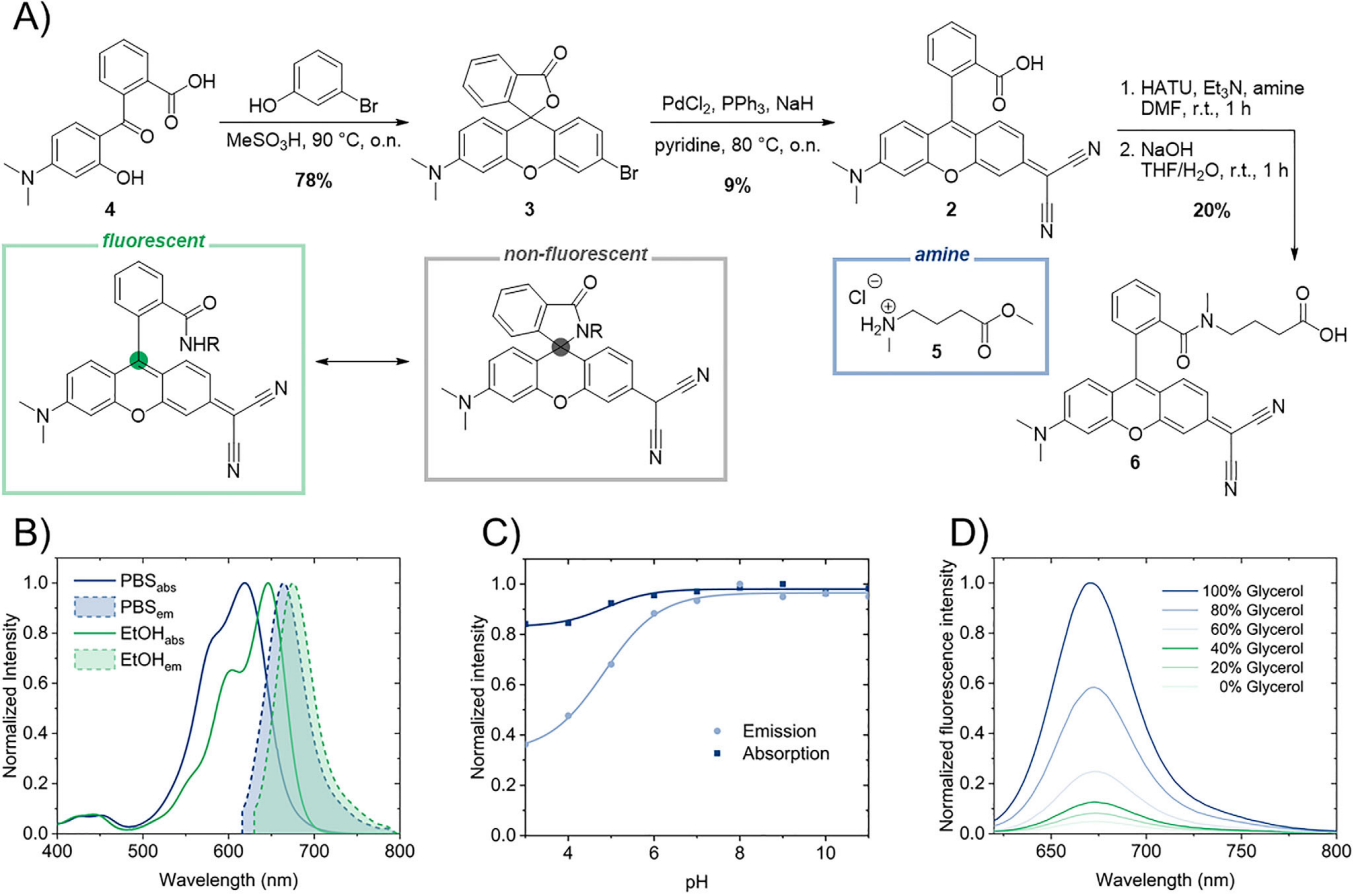
(A) Synthetic route of RhodaMal 620 starting from literature known keto acid **4** with open (fluorescent) and closed, spiro‐lactame (non‐fluorescent) equilibrium known for secondary amides. (B) Normalized absorption and emission spectra in PBS (pH 7.4, blue) and EtOH (green). (C) Normalized absorption (dark blue) and emission (light blue) intensities at different pH values. (D) Normalized fluorescence spectra at different glycerol/MeOH concentrations. Spectra and data points given are single measurements.

With dye **6** in hand, we examined the photophysical properties in a variety of solvents, the results are summarized in Table 1. In phosphate‐buffered saline (PBS, simulating physiological conditions) the dye exhibits absorption and emission maxima of 620 and 664 nm, respectively, corresponding to a 44 nm stokes shift (Figure 3B). The extinction coefficient is moderately high at 55,800 M^−1^ cm^−1^, whereas the fluorescence quantum yield is relatively low at 1%. In organic solvents, the dye displays slight bathochromic shifts and an increased extinction coefficient, reaching up to 82,600 M^−1^ cm^−1^ in MeOH (see Supporting Information). Notably, no enhancement in fluorescence quantum yield is observed, even in less polar environments where an increase is typically observed [28]. A minor increase of quantum yield to 2% was observed when measuring in sodium dodecyl sulfate (SDS) containing water. Other malononitrile donor–acceptor dyes have demonstrated substantial fluorescence increase in highly viscous environments, such as glycerol [24]. To test whether this effect applies to dye **6**, we measured the fluorescence intensity in different mixtures of methanol and glycerol. As visible in Figure 3D, a 20‐fold increase in fluorescence intensity is observed when transitioning from pure methanol to pure glycerol. Examining the photophysical properties in highly viscous media such as PEG 400 and glycerol showed an increased quantum yield up to 4% and 11%, respectively, while exhibiting high extinction coefficients of up to 90,800 observed for PEG 400. We furthermore incubated dye **6** in PBS at 37°C for up to 24 h to assess its aqueous stability and measured the absorption and emission over time. No major loss of absorption or emission intensity was observed, indicating high stability and hydrolytic resistance under these conditions (Figures S13 and S14). As shown by the pH‐dependent absorption and emission intensities (Figures 3C and S15), both maxima are reached at pH values above 7. The moderate decrease in absorbance at lower pH values presumably results with the formation of a protonated dye species with a reduced molar extinction coefficient, as no isosbestic point is visible in that pH range. The substantially stronger decrease in emission compared to the absorption indicates that the protonated species retains ground‐state absorption but exhibits a markedly reduced fluorescence quantum yield. Protonation most likely occurs at the electron‐rich alkylamine donor moiety, as the fluorescence intensity decreases over the same pH range. We reason that the dye emits from a charge‐seperated state, a common observation for push–pull chromophores [29]. Therefore, protonation of the donor moiety disturbs the emissive state and promotes non‐radiative deexcitation pathways.

**TABLE 1:** Summary of photophysical properties of RhodaMal 620 (**6**) in different solvents. Relative quantum yield determined using Oxazine 1 in Ethanol (*Φ* = 0.15) as reference [27]. All values given are after single measurements, except for quantum yield measurements (mean of *n* = 3).

Solvent	*λ*_abs_ (nm)	*λ*_em_ (nm)	Δ*λ* (nm)	*ε* (M^−1^ cm^−1^)	*Φ*_Fl_
PBS	620	664	44	55,800	0.01
MeOH	639	675	36	82,600	<0.01
EtOH	646	676	30	82,500	0.01
MeCN	643	674	31	81,800	0.01
DCM	656	683	27	72,400	0.01
H_2_O (20 mM SDS)	642	672	30	74,400	0.02
PEG 400	656	678	22	90,800	0.04
Glycerol	640	669	39	84,300	0.11

Given the slightly basic pH of the mitochondrial matrix (approximately pH 8), we identified mitochondrial targeting as a suitable model system for further live‐cell studies [30]. As the dye may induce cytotoxic effects and influence imaging results, we investigated HeLa cell viability using a Cell Counting Kit‐8 (CCK‐8) assay of the core dye structure (**6**). We observed the absence of any toxicity in concentrations up to 10 µM over 24 h of incubation at 37°C (Figure S19). These loadings far exceed concentrations usually used in live cell imaging experiments of ∼1 µM. We therefore conjugated TPP^+^ to **6**, yielding Mito‐Dye **6a** (Figure 4A). TPP^+^ conjugates accumulate and reside in mitochondria, driven by the negative mitochondrial potential. This accumulation however, can be influenced by release of membrane potential by the addition of uncouplers such as FCCP (carbonyl cyanide 4‐(trifluoromethoxy)‐phenylhydrazone) [29]. We then conducted live‐cell imaging experiments in HeLa cells using Mito‐Dye **6a**. To confirm mitochondrial targeting, we performed colocalization studies with MitoTracker Green, using ImageJ's JaCoP Plugin, with ROI's of full channel windows [31, 32]. As anticipated, there was substantial overlap between the two fluorescence channels, with a Pearson's correlation coefficient of 0.89, confirming efficient mitochondrial labelling by the TPP^+^‐conjugated dye (Figure 4B). Despite the dye's low quantum yield in previous photophysical experiments, clear mitochondrial imaging was achieved at 1 µM concentration of **6a**, which is typical concentration for small molecule probes [33]. We believe that the rigid and viscous environment of the mitochondrial matrix being rich in proteins, enzymes, and nucleic acids all involved in energy production and cellular signalling restricts non‐radiative decay pathways, which enhances the fluorescence signal through a locally increased viscosity [34]. Intrigued by the dye's performance in confocal imaging, we wanted to explore and evaluate **6a** for super‐resolution microscopy using time‐gated stimulated emission depletion (gSTED) in live HeLa cells at 1 µM concentration. As shown in Figure 4C, confocal images revealed homogenous staining of mitochondria but lacked morphological detail. In contrast, the gSTED and especially the deconvoluted images (noted as STED+) resolved the ladder‐like architecture of mitochondrial cristae, achieving subdiffraction resolution, with full width at half maximum (FWHM) values down to 77 nm in raw STED images, showcasing significant increase in resolution and their applicability in super‐resolution nanoscopy (Figure 4D,E). While recent studies achieved mitochondrial STED imaging with resolutions down to 35 nm (Yang et al.), several other mitochondrial probes have been reported with FWHM values in the range of 60 (Wang et al.) and 80 nm (Song et al.). Thus, the RhodaMal scaffold does not outperform established STED dyes, but places it within contemporary dyes for mitochondrial imaging [35, 36, 37, 38]. Unfortunately, significant fluorescence loss is observed and only 20% of fluorescence is achieved after repeated STED images, demonstrating rather mediocre photostability of the dye (Figure S18) in a biological environment.

**FIGURE 4:**
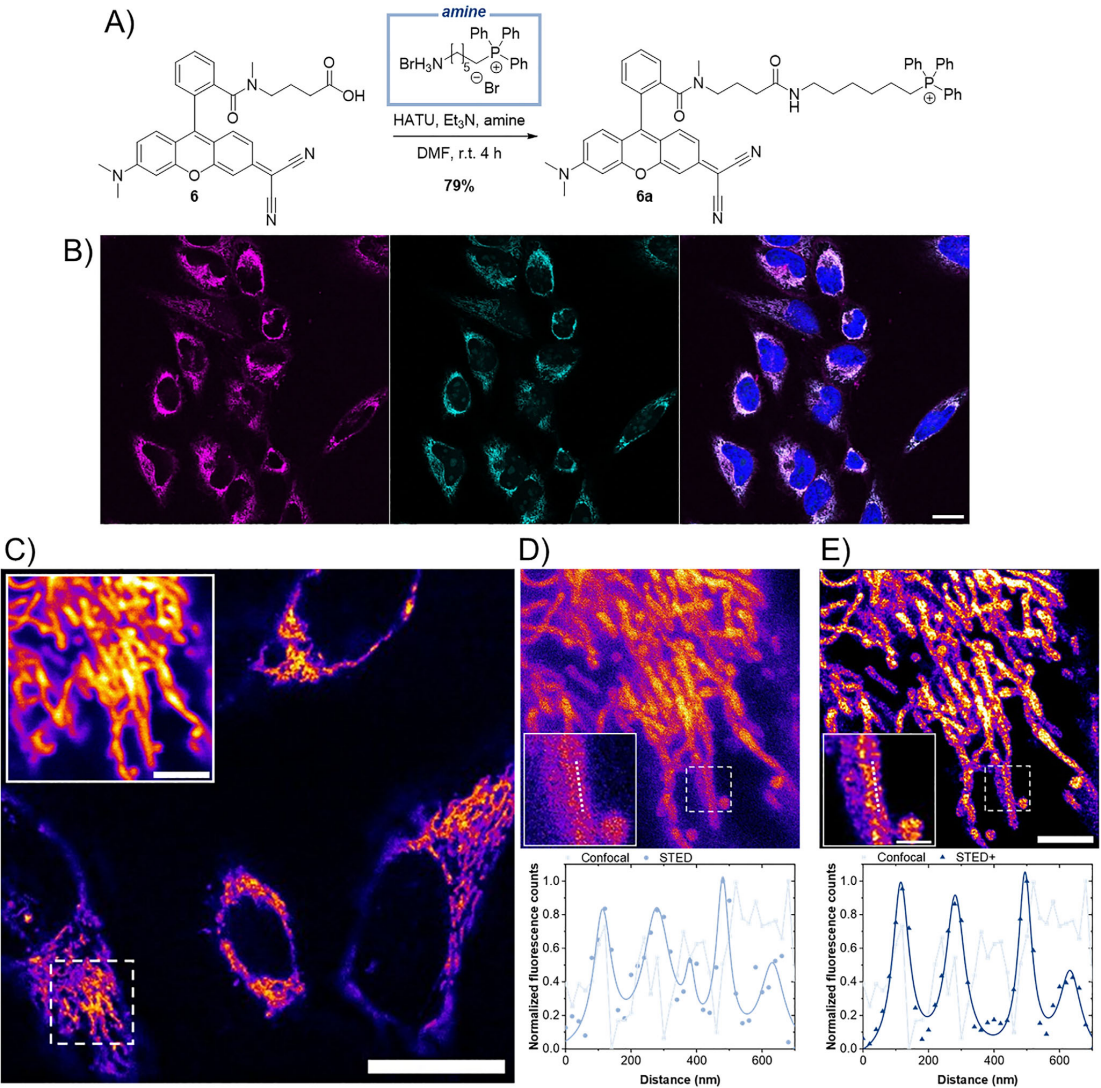
(A) Synthesis of Mito‐Dye **6a**. (B) Colocalization experiments using Mito‐Dye **6a** (left, magenta) and MitoTracker Green (middle, cyan) and merged channels (right) with blue = Hoechst 33258. (C–E) Live‐cell imaging with confocal (C), STED (D) and STED+(E) images and the respective fluorescence intensity profiles across dashed lines (700 nm). Scale bars: (B) 20 µm, (C) 20 µm with inset 2 µm, (D and E) 2 µm with insets 500 nm.

To address the issue of photostability, we pursued structural modifications inspired by Zhang and coworkers, who demonstrated that introducing a second amine functionality at the donor core enhances photo‐resistance by rigidizing the molecular structure [39]. We identified aryl bromide **7** as a suitable substrate for the palladium‐catalyzed introduction of malononitrile, which in turn should be obtained via the condensation of the analogous keto acid **8**. (Figure 5B). We investigated the Friedel–Crafts reaction using literature known tetrahydroquinoxaline **9**. After extensive screening of reaction conditions, we found that excess AlCl_3_ under mild heating furnished **8** in modest yield. However, ^1^H NMR analysis suggested partial demethylation of the methoxy group, resulting in a complex product mixture that proved unexpectedly difficult to separate. With the product mixture in hand, subsequent condensation with *m*‐bromophenol under harsh acidic conditions furnished bromide **7**, yet reproducibility on larger scales and purification appeared inconsistent and tedious. To circumvent these problems, we pivoted to the corresponding triflate **11** as an alternative substrate. This was easily accessible by triflating phenolic **10**. Aryltriflate **11** is also a promising substrate and should open up new chemical space, offering a streamlined route to otherwise inaccessible asymmetric rhodamines or recently developed N‐Cyano rhodamines [40]. While we endeavored these synthetic steps, we serendipitously came across of nucleophilic aromatic substitutions of dichloro fluorescins using LDA and related nitriles [41]. Intrigued by these results, we initially tested similar conditions to omit the use of palladium and were pleasantly surprised. Reacting **11** with malononitrile and LDA furnished **12** in satisfactory yield, bypassing the use of palladium. Similar conditions were examined for analogues triflate **13**, affording **2** in 20% yield (see Supporting Information). Again, converting the carboxylic acid of **12** to the tertiary amide and subsequent saponification led to RhodaMal 638 **14**, which was used for further photophysical studies. As seen in Table 2, a slight bathochromic shift can be observed for the absorption maximum (18 nm) and emission maximum (44 nm) compared to **6** in PBS, resulting in a Stokes shift of 70 nm. In PBS, the extinction coefficient decreases significantly to 26,400 M^−1^ cm^−1^, while the quantum yield resides at 0.01, similar to RhodaMal 620 **6**. In organic solvents, however, the extinction coefficient remains consistently high around 70,000 M^−1^ cm^−1^ in all tested solvents and the quantum yield increases to 5% in EtOH. This enhancement in quantum yield can be attributed to reduced twisted intramolecular charge transfer (TICT), facilitated by the structural rigidity of the tetrahydroquinoxaline moiety [42]. We could not evaluate the photophysical properties in SDS containing water, as the dye readily decomposed in this environment. Interestingly, for highly viscous media, the increase in quantum yield is less pronounced compared to **6**, while the extinction coefficient even decreases in these solvents compared to organic environments. The pH‐dependent profiles reveal a slight shift of their maxima toward more basic values relative to **6**. A substantial influence on the emission is observed, where it reaches only 20% in acidic environment (Figure 5D). In accordance with previous observations for dye **6**, we reason that the same rationale applies for dye **14**, although protonation is expected to have a stronger influence in this case, because the dye contains a more electron‐rich donor core bearing the two aminoalkyl substituents. We further evaluated the photostability of RhodaMal 638 in comparison to RhodaMal 620 and other fluorophores absorbing in the same spectral region, namely Cy5 and Tetramethylcarborhodamine, with the latter being a widely used dye in STED microscopy (Figure S17) [13, 43]. After irradiation with a 40 W Hg lamp for 10 min, both Cy5 and RhodaMal 620 have their fluorescence intensity drastically reduced to 10% and 22%, respectively (Figures 5E and S17). In contrast, RhodaMal 638 demonstrated a 3‐fold improved stability and retained 64% of its initial intensity after 10 min, proving enhanced photostability due to the incorporation of the second amine functionality. Carborhodamine retained 85% of its intensity under these conditions. Again, we additionally performed cell viability on HeLa cells and did not observe any toxicity of concentrations up to 10 µM for **14** (Figure S19). Encouraged by the improved photostability, we analogously conjugated RhodaMal 638 to TPP^+^ to obtain Mito‐Dye **14a** (see Supporting Information) and conducted live cell experiments in HeLa cells. We again initially performed colocalization experiments using MitoTracker Green and demonstrated substantial colocalization, proving efficient targeting mitochondria, with a Pearson's correlation coefficient of 0.88 (Figure 5F). Based on these promising results, we proceeded to perform live‐cell gSTED experiments again with a 640 nm excitation and 775 nm depletion laser. Unfortunately, no STED images could be obtained, as the absorption of RhodaMal 638 appears to partially overlap with the 775 nm depletion laser. This spectral overlap resulted in an instantaneous and irreversible photobleaching of the fluorophore under the irradiation conditions used in the miscroscope.

**FIGURE 5:**
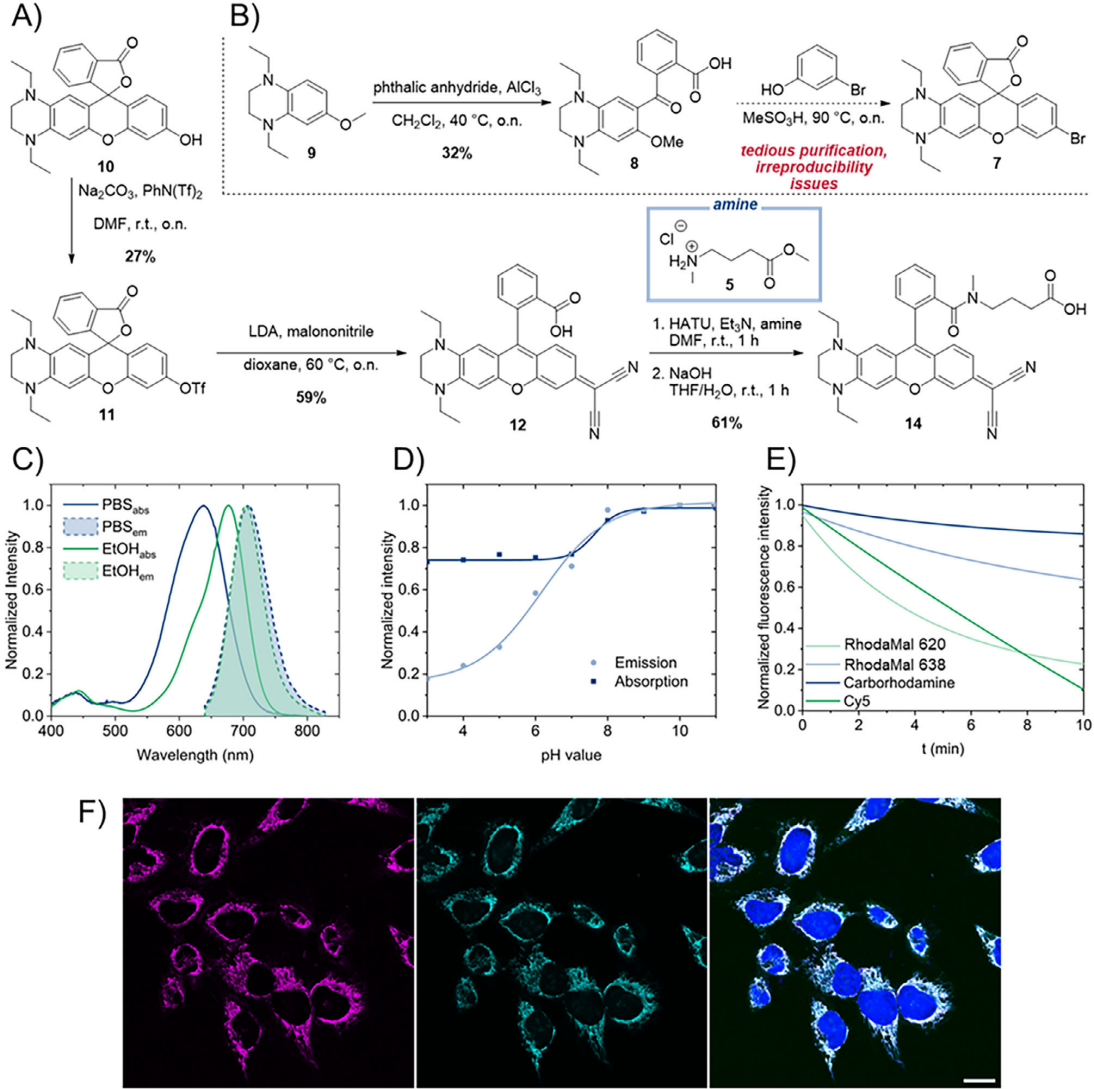
(A) Synthesis toward RhodaMal 638. (B) Initial strategy for the synthesis of aryl bromide **7**. (C) Normalized absorption and emission spectra in PBS (pH 7.4, blue) and EtOH (green). (D) Normalized absorption (dark blue) and emission (light blue) intensities at different pH values. (E) Normalized time‐dependent fluorescence intensity changes of RhodaMal 620, RhodaMal 638, Cy5, and Carborhodamine in PBS (pH = 7.4) after irradiation by a 40 W Hg lamp for 10 min. (F) Colocalization experiments using Mito‐Dye **14a** (left, magenta) and MitoTracker Green (middle, cyan) and merged channels (right) with blue = Hoechst 33258. Scale bar: 20 µm. Spectra and data points given are single measurements.

**TABLE 2:** Summary of photophysical properties of RhodaMal 638 (**14**) in different solvents. Relative quantum yield determined using Oxazine 1 in Ethanol (*Φ* = 0.15) as reference. All values given are after single measurements, except for quantum yield measurements (mean of *n* = 3).

Solvent	*λ*_abs_ (nm)	*λ*_em_ (nm)	Δ*λ* (nm)	*ε* (M^−1^ cm^−1^)	*Φ*_Fl_
PBS	638	708	70	26,400	0.01
MeOH	667	716	49	67,500	0.02
EtOH	677	707	30	72,700	0.05
MeCN	675	718	43	70,100	0.03
DCM	691	728	37	71,200	0.04
H2O (20 mM SDS)	n.d.	n.d.	n.d.	n.d.	n.d.
PEG400	689	724	35	58,200	0.05
Glycerol	666	712	46	62,200	0.06

## 3 Conclusion

In this work, we successfully developed two new cell permeable and red emitting dyes, RhodaMal 620 and RhodaMal 638, by incorporating malononitrile into the traditional rhodol scaffold. This modification resulted in pronounced bathochromic shifts, with absorption maxima at 620 and 638 nm and emission maxima at 664 and 708 nm, respectively, in PBS. Although both dyes exhibit rather low quantum yields in most solvents, we showed that increasing medium viscosity led to a dramatic, 20‐fold enhancement in fluorescence intensity. This suggests that their performance in live‐cell conditions, particularly within organelles such as mitochondria, can be substantially improved by the local microenvironment. Live‐cell confocal microscopy in HeLa cells confirmed efficient and selective mitochondrial staining of the TPP^+^ conjugates, as demonstrated by strong colocalization with MitoTracker Green (with Pearson's correlation coefficients up to 0.89). Notably, Mito‐Dye **6a** enabled super‐resolution imaging of mitochondrial structures using gSTED microscopy, revealing details of the mitochondrial cristae architecture with raw FWHM of 77 nm. Our dyes expand the palette of far‐red fluorophores available for live‐cell imaging and further strategies to improve the stability of RhodaMals while matching spectral properties for commercial STED microscope setups are underway in our laboratories.

## Funding

This work was supported by Deutsche Forschungsgemeinschaft (Core Facility BioSupraMol), Dahlem Research School, Freie Universität Berlin.

## Conflicts of Interest

The authors declare no conflicts of interest.

## Use of Generative AI and AI‐Assisted Technologies in the Writing Process

During the preparation of this article, the authors used AI‐assisted technology (ChatGPT and OpenAI) to improve grammar, spelling, and readability. The authors reviewed and edited the output as needed and take full responsibility for the content of the article. No AI tools were used for data analysis, data interpretation, or figure generation.

## Supporting information



The authors have cited additional references within the Supporting Information [44, 45].
**Supporting File 1**: chem71410‐sup‐0001‐SuppMat.docx

## Data Availability

The data that support the findings of this study are available in the Supporting Information of this article.
