# Supplementary material for: RhodaMal Dyes: Novel Red Emissive Fluorophores as Probes for High Contrast Live‐Cell Imaging
Source: Chemistry. 2026 Jul 18;32(39):e71410. doi: 10.1002/chem.71410 (PMC13715048; doi:10.1002/chem.71410)
Supplement: Supplementary file 1 [file CHEM-32-e71410-s001.docx]

Chemistry–A European Journal

Supporting Information

**RhodaMal Dyes: Novel Red Emissive Fluorophores as Probes for High Contrast Live-Cell Imaging**

Alexandros Mavroskoufis^[a]^, Paul Aßmann^[a]^, L.M. Filla^[a]^, Iulia Bilici^[a]^, Elisa Quaas^[b]^, Marta Maglione^[b]^, Stephan Sigrist^[c]^, Rainer Haag^[ab]^, Kai Licha^[a]*^

Contents

[1. General Information	3](#_Toc219106756)

[1.1 Materials and Methods	3](#_Toc219106757)

[2. DFT calculations.	5](#_Toc219106758)

[3. Photophysical studies	7](#_Toc219106759)

[4. Synthetic procedures	14](#_Toc219106760)

[5. Spectra	21](#_Toc219106761)

[6. References	29](#_Toc219106762)

# 1. General Information

## 1.1 Materials and Methods

Reactions with air or moisture sensitive substances were carried out under an argon atmosphere using standard Schlenk technique. Ambient or room temperature (r.t.) refers to 18–23 °C. Heating of reactions was performed with an oil bath unless otherwise noted.

Unless otherwise noted, all staring materials and reagents were purchased from commercial distributors and used without further purification. Anhydrous dichloromethane (DCM), tetrahydrofurane (THF), *N*,*N*-dimethylformamide (DMF), pyridine were purchased from Sigma Aldrich. Triethylamine was distilled from calcium hydride and stored under argon over KOH. Solvents for automated column chromatography were purchased in technical grade (Cyclohexane, MeOH) and HPLC grade (EtOAc and DCM).

The following compounds were prepared according to literature known procedures: Acid **4^[1]^**, tetrahydrochinoxaline **9^[2]^**, dye **10^[3]^**.

**Reaction monitoring:** Reactions were monitored by thin layer chromatography (TLC). TLC-analysis was performed on silica gel coated aluminum plates ALUGRAM® Xtra SIL G/UV_254_ purchased from MACHEREYNAGEL. Products were visualized by UV light at 254 nm and by using staining reagents (based on KMnO_4_ and Ceric Ammonium Molybdate).

**NMR spectsocopy:** ^1^H and ^13^C NMR spectra in solution were measured with the spectrometers ECX400 (400 MHz) ECP500 (500 MHz) and ECZ600 (600 MHz) from JEOL and Avance500 (500 MHz) and Avance700 (700 MHz) from Bruker. The chemical shifts (δ) are listed in parts per million (ppm) and are reported relative to the corresponding residual nondeuterated solvent signal (e.g. CDCl_3_: δ_H_ = 7.26 ppm, δ_C_ = 77.16 ppm). Coupling constants (*J*) are given in Hz. Multiplicity is indicated as follows: s (singlet), d (doublet), t (triplet), q (quartet), br = broad and combinations thereof. In the case where no multiplicity could be identified, the chemical shift range of the signal is given as m (multiplet). ^13^C NMR spectra are ^1^H-broadband decoupled.

**High resolution mass spectrometry:** High resolution mass spectra (HRMS) were measured with an Agilent 6210 ESI-TOF (10 μL/min, 1.0 bar, 4 kV) instrument.

**Chromatography:** NP automated column chromatography was done on a CombiFlash Rf (Teledyne ISCO) using prepacked silica columns (30 μm). The crude product was preloaded on Isolute® or Celite®. Analytical HPLC was performed with a Smartline system from Knauer (Berlin, Germany), equipped with dual pumps 1000, a degasser, an autosampler 3950 and variable wavelength UV detector 2500. Preparative HPLC was performed on a high-pressure gradient system (stainless steel), equipped with a Shimadzu LC-8A pump, Shimadzu CBM-20A controller, variable wavelength UV detector from Knauer and a Rheodyne injector with 4 mL sample loop

**UV/Vis and fluorescence spectroscopy:** UV/Vis spectra were recorded using an Agilent Technologies Cary 8454 UV/VIS spectrometer and fluorescence spectra using a JASCO FP6500 spectrometer. In a typical experiment, a dye stock solution of 1 mM was prepared using dimethyl sulfoxide (DMSO). For the actual measurement, the concentration was diluted so the DMSO content did not exceed 1% (v/v) yielding a volume of 1 mL or 2 mL in a cuvette. Measurements were performed at ambient temperature. Phosphate-buffered saline (PBS) was at pH = 7.4 unless otherwise noted. For quantitative measurements, 1 cm path length, 3.5 mL quartz cuvettes from Hellma® were used. For qualitative measurements, UV-cuvette semi-micro type cuvettes from BRAND GmbH & Co. KG were used. Extinction coefficients were calculated using Lambert Beer Law.

**Photostability studies:**

Photostability studies were conducted using an Ushio USH-102D Hg lamp, a LOT LSH102 housing connected to a LOT LSN150 universal power supply. For the experiment, after thermal balance of the Hg-lamp, the sample was placed in a pre-marked spot, stirred and irradiated at 5 uM in PBS (pH = 7.4) at ambient temperature and measured at different timepoints. Fluorescence spectra were integrated and normalized to evaluate the photostability.

**Cell culture**

HeLa cells were obtained from Leibniz-Institut DSMZ and cultured in DMEM Medium supplemented with 10% (v/v) FBS, 100 U/mL penicillin and 100 μg/mL streptomycin at 37°C and 5% CO2. Cells were seeded in 8-well slides (Ibidi GmbH, Gräfelfing, Germany) at a density of 5 x 104 cells/ml and incubated overnight for cell attachment. Then sample (solved in deionized water) was added to cells at different concentrations. After incubation of 2h cells were stained with Hoechst 33342 dye (1 μg/mL, Life Technologies GmbH, Darmstadt, Germany), or additionally with MitoTracker Green (100 nM), washed 3 times with PBS and covered with fresh DMEM cell culture medium.

**Cell Viability**

The cell viability was investigated on Hela cells (ATCC number CCL-2). The cells were cultured in Dulbecco’s Modified Eagle’s Medium (DMEM) supplemented with 10% FBS, 1% penicillin and streptomycin (all from Gibco BRL, Eggenstein, Germany). The cells were subcultured twice a week when they reached 90% confluency. For the cell viability assay, 100 µL of a cell suspension in DMEM containing 1×104 cells/mL were seeded in each inner well of a 96-well plate and incubated at 37 °C and 5% CO2 till confluency was observed. The outer wells were prepared with only 100 µL DMEM. The stock solutions of the samples (1 mM in DMSO) were 100-fold diluted in the cell culture medium. A two-fold dilution series was performed. After reaching confluency, the medium was removed and 100 µL per concentration of the desired compound was added to the cells in triplicates and in addition to one outer well for the background correction. SDS (1 w%) and DMSO (1:100) served as controls. The cells together with the compounds were incubated for 24 h at 37 °C and 5% CO2. Afterwards the CCK-8 solution was added (10 µL/well), and the absorbance at a measurement wavelength of 450 nm and a reference wavelength of 650 nm was measured after 2 h incubation using a Tecan plate reader. Measurements were performed in triplicates. The cell viability was calculated by normalizing to the non-treated control after correction by the background using the Excel software. The graphs were plotted in GraphPad Prim 6.

**Confocal and STED setups**

Confocal images were taken with an inverted confocal laser scanning microscope Leica DMI6000CSB SP8 (Leica, Wetzlar, Germany) with a 63x/1.4 HC PL APO CS2 oil immersion objective using the manufacture given LAS X software in sequential mode with the following channel settings: Transmission channel (grey intensity values): excitation laser line 405 nm, detection of transmitted light (photomultiplier: PMT); Mito-Dye channel: excitation laser line 633 nm, detection range 640 nm–750 nm (PMT); Hoechst channel: excitation laser line 405 nm, detection range 409 nm–495 nm (HyD); Mito Tracker channel: excitation laser line 561 nm, detection range 567 nm–627 nm (PMT).

2D gSTED microscopy was performed using an Abberior Instruments Expert Line STED setup equipped with an inverted IX83 microscope (Olympus, Japan), two pulsed STED lasers for depletion at 775 nm (0.98 ns pulse duration, up to 80 MHz repetition rate) and at 595 nm (0.52 ns pulse duration, 40 MHz repetition rate) and pulsed excitation lasers (at 488 nm, 561 nm and 640 nm). Confocal and multi-channel gSTED scanning was performed with 100× oil-immersion objective lens (Olympus, NA=1.4). The dye was excited at 640 nm and depleted with a pulsed STED laser at 775 nm, time gating was set to 550 ps. Fluorescence signals were detected sequentially by line by avalanche photodiode detectors at appropriate spectral regions. 2D gSTED and confocal images were acquired with a pixel dwell time of 2 µs, with 30x lines accumulation and 10x line accumulation, respectively, at 16bit sampling and a field of view of 10µm x 10µm. Lateral pixel size was set to 20 nm. Raw gSTED images were processed for Richardson-Lucy deconvolution using the Imspector software (version 16.3.15507, Abberior Instruments, Germany). The point spread function was automatically computed with a 2D Lorentz function having a full- width half- maximum of 40 nm, based on measurements with 40 nm crimson beads. Default deconvolution settings were applied. To determine the FWHM, a polymodal Lorentzian line profile was applied at the shown dashed lines (see Figure 4 in manuscript).

# 2. DFT calculations.

Table S1: The Cartesian (XYZ) coordinates of the optimized geometry of **1**.

C	0.000000	0.000000	0.000000

N	1.266904	0.033653	-0.719248

C	1.595694	-1.043356	-1.643631

H	1.684906	-0.680457	-2.677033

H	2.538849	-1.534347	-1.369677

H	0.804907	-1.793486	-1.614983

C	2.126808	1.095225	-0.564794

C	1.818496	2.172902	0.318223

C	2.690013	3.234125	0.472735

C	3.917668	3.308114	-0.224782

C	4.203194	2.237075	-1.102451

C	3.345559	1.157800	-1.277458

H	3.647653	0.385538	-1.972468

O	5.351508	2.213929	-1.834188

C	6.270339	3.235887	-1.761045

C	6.021776	4.356567	-0.871914

C	4.872103	4.379222	-0.105619

C	4.553435	5.545020	0.779668

C	4.738584	5.571300	2.182765

C	4.355221	6.710610	2.912837

C	3.802656	7.820542	2.280393

C	3.621040	7.801328	0.894965

C	3.990292	6.673477	0.160542

H	3.841544	6.656284	-0.915250

H	3.192891	8.659296	0.384125

H	3.518610	8.692504	2.861992

H	4.507691	6.698924	3.986806

C	5.332097	4.464987	2.997655

O	5.901513	3.476163	2.272919

H	6.249053	2.828856	2.910198

O	5.326281	4.443372	4.215513

C	7.045610	5.379382	-0.835301

C	8.162998	5.300264	-1.600024

C	8.410165	4.168227	-2.514327

C	7.382871	3.134687	-2.539119

H	7.526987	2.279583	-3.190752

O	9.435141	4.112250	-3.213834

H	8.928266	6.070331	-1.567782

H	6.897159	6.223695	-0.169040

H	2.420556	4.037747	1.150616

H	0.892902	2.173624	0.879420

H	0.151029	0.014911	1.087468

H	-0.646305	0.846577	-0.269885

H	-0.528491	-0.920053	-0.251663

Table S2: The Cartesian (XYZ) coordinates of the optimized geometry of **2**.

C	0.000000	0.000000	0.000000

N	1.275366	-0.665621	-0.242412

C	2.410764	0.067828	-0.472408

C	2.380825	1.496875	-0.469826

C	3.526404	2.231430	-0.695566

C	4.779672	1.616837	-0.937954

C	4.787995	0.199998	-0.946950

C	3.651250	-0.563451	-0.722964

H	3.755741	-1.639966	-0.750153

O	5.937144	-0.492835	-1.184120

C	7.118927	0.154142	-1.433375

C	7.165454	1.595462	-1.429657

C	6.006850	2.318865	-1.170086

C	6.005161	3.815267	-1.217087

C	6.024657	4.658577	-0.080475

C	5.964909	6.053042	-0.250282

C	5.898305	6.622766	-1.518755

C	5.885705	5.794771	-2.644163

C	5.934888	4.408482	-2.488627

H	5.914655	3.765500	-3.363867

H	5.836581	6.222319	-3.641602

H	5.859658	7.702391	-1.628289

H	5.977588	6.673265	0.639698

C	6.114547	4.192399	1.338952

O	5.931845	4.914569	2.301586

O	6.439395	2.886748	1.480070

H	6.475338	2.710061	2.435963

C	8.456801	2.180625	-1.680472

C	9.556868	1.414801	-1.922639

C	9.485935	-0.029658	-1.935752

C	8.215371	-0.629138	-1.675101

H	8.108746	-1.708492	-1.665455

C	10.632197	-0.805745	-2.193222

C	11.894417	-0.204194	-2.453779

N	12.921445	0.310064	-2.665979

C	10.570114	-2.226467	-2.203798

N	10.486503	-3.391476	-2.205597

H	10.515653	1.888213	-2.107988

H	8.544991	3.261852	-1.672265

H	3.465546	3.314877	-0.689318

H	1.451987	2.023086	-0.291293

C	1.329303	-2.122957	-0.265218

H	1.645700	-2.501153	-1.246554

H	2.020819	-2.511412	0.493792

H	0.337170	-2.521500	-0.052800

H	-0.311224	0.609120	-0.859166

H	-0.767339	-0.754725	0.172991

H	0.043651	0.646204	0.886092

# 3. Photophysical studies

Figure S1: Normalized absorption and emission spectra of RhodaMal 620 in different solvents.

Figure S2: Normalized absorption and emission spectra of RhodaMal 638 in different solvents

Figure S3: UV-Vis spectra of RhodaMal 620 in DCM at different concentrations with linear dependence of absorbance at λ_max._

Figure S4: UV-Vis spectra of RhodaMal 620 in EtOH at different concentrations with linear dependence of absorbance at λ_max_.

Figure S5: UV-Vis spectra of RhodaMal 620 in MeCN at different concentrations with linear dependence of absorbance at λ_max_.

Figure S6: UV-Vis spectra of RhodaMal 620 in MeOH at different concentrations with linear dependence of absorbance at λ_max_.

Figure S7: UV-Vis spectra of RhodaMal 620 in PBS (pH = 7.4) at different concentrations with linear dependence of absorbance at λ_max_.

Figure S8: UV-Vis spectra of RhodaMal 638 in DCM at different concentrations with linear dependence of absorbance at λ_max_.

Figure S9: UV-Vis spectra of RhodaMal 638 in EtOH at different concentrations with linear dependence of absorbance at λ_max_.

Figure S10: UV-Vis spectra of RhodaMal 638 in MeCN at different concentrations with linear dependence of absorbance at λ_max_.

Figure S11: UV-Vis spectra of RhodaMal 638 in MeOH at different concentrations with linear dependence of absorbance at λ_max_.

Figure S12: UV-Vis spectra of RhodaMal 638 in PBS (pH = 7.4) at different concentrations with linear dependence of absorbance at λ_max_ at 2.5 μM (638 nm) and λ_max_ at 10 μM (609 nm).

Figure S13: Normalized absorption spectra of RhodaMal 620 in PBS at 37 °C at different timepoints.

Figure S14: Normalized emission spectra of RhodaMal 620 in PBS at 37 °C at different timepoints.

Figure S15: Absorption (left) and emission (right) of RhodaMal 620 at different pH values.

Figure S16: Absorption (left) and emission (right) of RhodaMal 638 at different pH values.

Figure S17: Fluorescence intensity of respective dye in PBS (pH = 7.4) at 5 μM concentration after irradiation with a 40 W Hg arc lamp.

Figure S18: Normalized fluorescence intensity of full channel window after several STED scans. Scale bar: 2 μm.

Figure S19: Cell viability assessment using cell counting kit 8 (CCK-8) on Hela cells. The samples were incubated for 24 h with the cells, the measurements were performed in triplicates and presented ±SD. No cytotoxicity was observed in the tested concentration regime for RhodaMal 638 and RhodaMal 620. SDS (1 w%) and DMSO (1:100) served as control experiments.

# 4. Synthetic procedures

Acid **4** (100 mg, 351 μmol, 1.0 equiv) and *m*-bromophenol (66.7 mg, 386 μmol, 1.1 equiv) were suspended in methane sulfonic acid (1.40 mL, 0.25m of **4**). The reaction mixture was set under argon atmosphere and stirred at 90 °C overnight. After cooling to ambient temperature, the mixture was poured on ice (25 mL) and saturated aqueous NaHCO_3_ solution (25 mL) was added. The aqueous phase was extracted with DCM (3 x 25 mL) and the combined organic phases were washed with brine (25 mL), dried over anhydrous Na_2_SO_4_ and the solvent was removed under reduced pressure. The crude product was purified by automated column chromatography (SiO_2_, CyH/EtOAc 0 to 100%, linear gradient) to afford aryl bromide **3** (116 mg, 275 μmol, 78%) as a pink solid.

**^1^H NMR** (600 MHz, CD_2_Cl_2_): δ = 7.99 (dt, J = 7.6, 0.9 Hz, 1H), 7.69 (td, J = 7.5, 1.2 Hz, 1H), 7.64 (td, J = 7.5, 1.0 Hz, 1H), 7.47 (d, J = 2.0 Hz, 1H), 7.15 (ddd, J = 8.5, 4.6, 1.4 Hz, 2H), 6.66 (d, J = 8.5 Hz, 1H), 6.61 (d, J = 8.8 Hz, 1H), 6.51 (d, J = 2.6 Hz, 1H), 6.45 (dd, J = 8.9, 2.6 Hz, 1H), 2.99 (s, 6H) ppm.

**^13^C NMR** (151 MHz, CD_2_Cl_2_): δ = 169.5, 153.2, 152.7, 152.6, 152.5, 135.4, 130.2, 129.8, 128.8, 127.1, 126.9, 125.2, 124.2, 123.8, 120.5, 119.3, 109.6, 105.8, 98.6, 83.3, 40.4 ppm.

**HRMS** (ESI, pos.): *m/z* calc for C_22_H_16_BrNO_3_Na^+^ [M+Na^+^]: 444.02061, found 444.0179.

**2** was synthesized according to the literature-known procedure. The precatalyst was prepared by mixing PdCl_2_ (2.10 mg, 11.8   μmol, 5 mol%) and PPh_3_ (9.32 mg, 35.5  μmol, 15 mol%) in pyridine (200  μL) which was stirred for 15 min at 60 °C. NaH (60% on mineral oil, 28.4 mg, 710  μmol, 3.0 equiv) was added to malononitrile (19.6 mg, 296  μmol, 1.25 equiv) dissolved in pyridine (500  μL) and stirred for 5 min at ambient temperature. Then, the precatalyst was added and the mixture was stirred for an additional 5 min. Aryl bromide **3** (100 mg, 237  μmol, 1.0 equiv) in pyridine (500  μL) was added and the reaction mixture was stirred at 80 °C overnight. After cooling to ambient temperature, the reaction was quenched with aqueous HCl (1m, 20 mL) and the aqueous phase was extracted with EtOAc (3 x 15 mL). The combined organic phases were washed with brine (25 mL), dried over anhydrous Na_2_SO_4_ and the solvent was removed under reduced pressure. The crude product was purified by automated column chromatography (SiO_2_, DCM/MeOH, 0 to 10%, linear gradient) and subsequent RP-HPLC (H_2_O/MeCN 50% with 0.1% formic acid additive) to afford dye **2** (8.30 mg, 20.4 μmol, 8.6%) as a blue solid after lyophilization.

**^1^H NMR** (700 MHz, DMSO-d6): δ = 8.16 (d, J = 7.9 Hz, 1H), 7.83 (td, J = 7.5, 1.2 Hz, 1H), 7.76 (td, J = 7.6, 1.2 Hz, 1H), 7.41 (d, J = 7.5 Hz, 1H), 6.87 (d, J = 9.2 Hz, 2H), 6.85 – 6.81 (m, 2H), 6.78 (d, J = 8.9 Hz, 1H), 6.71 (dz, J = 2.2 Hz, 1H), 3.15 (s, 6H) ppm.

**^13^C NMR** (176 MHz, DMSO-d6): δ = 166.8, 163.0, 155.2, 132.9, 130.5, 130.0, 129.6, 129.2, 119.7, 114.2, 112.8, 111.8, 101.3, 96.6, 79.1, 54.8, 40.1 ppm.

**HRMS** (ESI, neg.): *m/z* calc for C_25_H_16_N_3_O_3_^-^ [M-H+]^-^: 406.1197, found 406.1177.

Rhodol **1** (1.00 g, 2.73 mmol, 1.0 equiv) was dissolved in DMF (13 mL) at ambient temperature. N-phenyl-bis(trifluoromethane sulfonimide) (1.99 g, 5.57 mmol, 2.0 qeuiv) and Na_2_CO_3_ (1.47 g, 13.9 mmol, 5.0 equiv) were added subsequently and the reaction mixture was stirred overnight. The reaction was poured on ice cold water (100 mL) and extracted with EtOAc (2 x 50 mL), the combined organic phases were washed with a mixture of ice cold water and brine (4:1, 3 x 100 mL; note: brine was added to induce phase seperation), brine (50 mL), dried over anhydrous Na_2_SO_4_ and the solvent was removed under reduced pressure. The crude product was purified by automated column chromatography (SiO_2_, CyH/EtOAc 0 to 100%, linear gradient) to afford aryl triflate **11** (737 mg, 1.50 mmol, 54%) as a red solid.

**^1^H NMR** (600 MHz, CDCl_3_): δ = 8.03 (dt, J = 7.6, 1.0 Hz, 1H), 7.69 (td, J = 7.5, 1.2 Hz, 1H), 7.64 (td, J = 7.5, 1.0 Hz, 1H), 7.22 (d, J = 2.4 Hz, 1H), 7.18 (d, J = 7.6 Hz, 1H), 6.92 (dd, J = 8.8, 2.5 Hz, 1H), 6.87 (d, J = 8.8 Hz, 1H), 6.62 (d, J = 8.9 Hz, 1H), 6.50 (d, J = 2.6 Hz, 1H), 6.44 (dd, J = 8.9, 2.6 Hz, 1H), 3.00 (s, 6H) ppm.

**^19^F NMR** (564 MHz, CDCl_3_): δ = -72.56 ppm.

**HRMS** (ESI, pos.): *m/z* calc for C_23_H_16_F_3_NO_6_SNa^+^ [M+Na^+^]: 514.0542, found 514.0586.

Diisopropylamine (32.2 μL, 23.1 mg, 228 μmol, 1.1 equiv) was dissolved in dioxane (2 mL). Then, *n*-BuLi (2.5m in hexanes, 91.3 μL, 228 μmol, 1.1 equiv) was slowly added and the mixture was stirred for 15 min. Malononitrile (20.6 mg, 311 μmol, 1.5 equiv) was added to the mixture which was stirred for an additional 15 min at ambient temperature. Triflate **13** (102 mg, 208 μmol, 1.0 equiv) was added and the reaction mixture was heated to 100 °C and overnight. After cooling to ambient temperature, the reaction was quenched with aqueous HCl (1m, 10 mL) and the aqueous phase was extracted with EtOAc (3 x 10 mL). The combined organic phases were washed with brine (10 mL), dried over anhydrous Na_2_SO_4_ and the solvent was removed under reduced pressure. The crude product was purified by automated column chromatography (SiO_2_, DCM/MeOH, 0 to 10%, linear gradient) and subsequent RP-HPLC (H_2_O/MeCN 50% with 0.1% formic acid additive) to afford dye **2** (16.3 mg, 129 μmol, 20%) as a blue solid.

See spectroscopic data above.

Dye **2** (52.7 mg, 129 μmol, 1.0 equiv) was dissolved in DMF (2 mL) at ambient temperature. The mixture was treated with Et₃N (108 μL, 78.5 mg, 776 μmol, 6.0 equiv) and HATU (6.39 mg, 168 μmol, 1.30 equiv) and stirred for 15 min. Then, amine **5** (32.5 mg, 194 μmol, 1.50 eq.) was added and the resulting solution was stirred for 40 min. The reaction was carefully quenched with saturated aqueous NaHCO_3_ (5 mL) and water (5 mL). The aqueous phase was extracted with DCM (3 x 10 mL), the combined organic phases were washed with brine (10 mL) and dried over anhydrous Na_2_SO_4_. The solvent was removed under reduced pressure and the crude product was purified by automated column chromatography (SiO_2_, CyH/acetone, 50%). The fractions containing the product were collected, the solvent was removed under reduced pressure and directly subjected to the subsequent saponification. The crude (43.6 mg) was dissolved in a mixture of THF/H_2_O (1:1.5, 20 mL) and NaOH (1M, 500 μL) was slowly added. The reaction mixture was stirred for 2 h and quenched by addition of glacial acidic acid (2 mL). The aqueous phase was extracted with DCM (3 x 10 mL), the combined organic phases were washed with brine (15 mL), dried over anhydrous Na_2_SO_4_ and the solvent was removed under recuced pressure. The crude product was purified RP-HPLC (H_2_O/MeCN 50% with 0.1% formic acid additive) to afford RhodaMal 620 **6** (21.3 mg, 42.0 μmol, 33% over two steps) as a blue solid after lyophilization.

**^1^H NMR** (700 MHz, DMSO-d6): δ = 7.72 – 7.66 (m, 2H), 7.65 – 7.60 (m, 1H, major), 7.60 – 7.55 (m, 1H, minor), 7.53 – 7.47 (m, 1H, major), 7.47 – 7.42 (m, 1H, minor), 7.05 – 7.00 (m, 1H), 7.00 – 6.93 (m, 3H), 6.87 (d, J = 2.4 Hz, 1H, minor), 6.84 (d, J = 2.4 Hz, 1H, major) , 6.71 (s, 1H), 3.20 (s, 6H), 3.15 – 3.10 (m, 2H), 2.80 (s, 3H, major), 2.60 (s, 3H, minor), 2.12 – 2.04 (m, 2H, minor), 1.81 – 1.67 (m, 2H, major), 1.67 – 1.53 (m, 2H, minor), 1.33 – 1.26 (m, 2H, major) ppm.

**^13^C NMR** (176 MHz, DMSO-d6, only major reported) δ 174.0, 167.5, 156.2, 155.8, 155.7, 154.8, 152.7, 136.5, 130.8, 130.4, 130.2, 130.2, 129.6, 129.2, 127.1, 120.2, 118.8, 118.6, 115.9, 113.1, 112.7, 101.0, 96.4, 69.7, 49.4, 45.6, 40.2, 36.9, 30.7, 21.4 ppm.

**HRMS** (ESI, pos.): *m/z* calc for C_30_H_26_N_4_O_4_Na^+^ [M+Na^+^]: 529.1846, found 529.1872.

RhodaMal 620 **6** (1.00 mg, 1.97 μmol, 1.0 equiv) was dissolved in DMF (200 μL). The mixture was treated with HATU (1m in DMF, 2.96 μL, 1.5 equiv) and Et_3_N (1m in DMF, 15.8 μL, 8.0 equiv) subsequently and stirred for 5 min. Then, amine (0.1m in DMF, 39.5 μL, 2.0 equiv) was added and the reaction mixture was stirred overnight. The solvent was removed under reduced pressure and the crude product was directly subjected to RP-HPLC (H2O/MeCN 30%, with 0.1% formic acid additive), yielding Mito-Dye **6a** (1.43 mg, 1.53 μmol, 79%) as dark blue solid after lyophilization.

**HRMS** (ESI, pos.): *m/z* calc for C_54_H_53_N_5_O_3_P^+^ [M]^+^: 850.3881, found 850.3950.

Dye **10** (500 mg, 1.17 mmol, 1.0 equiv) was dissolved in anhydrous DMF (13 mL) at ambient temperature. N-phenyl-bis(trifluoromethane sulfonimide) (834 mg, 2.33 mmol, 2.0 equiv) and Na_2_CO_3_ (618 mg, 5.84 mmol, 5.0 equiv) were added subsequently and the solution was stirred overnight. The reaction was poured on ice (20 mL) and H_2_O (30 mL) was added. The aqueous phase was extracted with EtOAc (2 x 50 mL) and the combined organic phases were washed with ice-cold water (3 x 50 mL), brine (50 mL) and dried over anhydrous Na_2_SO_4_. The solvent was removed under reduced pressure and the crude product was purified by automated column chromatography (SiO_2_, DCM/MeOH, 0 to 10%, linear gradient) to afford aryl triflate **11** (180 mg, 321 μmol, 28%) as a dark reddish solid.

**^1^H NMR** (600 MHz, CDCl_3_): δ = 8.03 (dd, J = 7.6, 1.0 Hz, 1H), 7.67 (td, J = 7.5, 1.2 Hz, 1H), 7.62 (td, J = 7.5, 1.0 Hz, 1H), 7.20 (dd, J = 7.7, 0.9 Hz, 1H), 7.18 (d, J = 2.4 Hz, 1H), 6.39 (s, 1H), 5.68 (s, 1H), 3.44 – 3.28 (m, 4H), 3.21 – 3.13 (m, 2H), 3.00 (ddd, J = 14.2, 7.1, 7.1 Hz, 1H), 2.93 (ddd, J = 14.2, 7.1, 7.1 Hz, 1H), 1.21 (t, J = 7.1 Hz, 3H), 0.86 (t, J = 7.1 Hz, 3H) ppm.

**^13^C NMR** (151 MHz, CDCl_3_): δ = 169.3, 152.8, 152.7, 149.8, 144.9, 138.6, 135.0, 132.4, 130.1, 129.8, 126.9, 125.1, 124.1, 119.8, 118.7 (q, *J* = 320.8 Hz), 115.8, 115.5, 110.2, 107.5, 104.4, 96.9, 84.3, 46.8, 45.5, 45.5, 45.4, 10.3, 9.6. ppm.

**^19^F NMR** (376 MHz, CDCl_3_): δ = -72.56 ppm.

**HRMS** (ESI, pos.): *m/z* calc for C_27_H_23_F_3_N_2_O_6_SH^+^ [M+H^+^]: 561.1302, found 561.1328; calc for C_27_H_23_F_3_N_2_O_6_SNa^+^ [M+Na^+^]: 583.1121, found 583.1138.

Diisopropylamine (54.2 μL, 39.0 mg, 385 μmol, 1.2 equiv) was dissolved in dioxane (2 mL). Then, *n*-BuLi (2.5m in hexanes, 141 μL, 353 μmol, 1.1 equiv) was slowly added and the mixture was stirred for 10 min. Malononitrile (31.8 mg, 382 μmol, 1.5 equiv) was added to the mixture which was stirred for an additional 15 min at ambient temperature. Triflate **11** (102 mg, 208 μmol, 1.0 equiv) was added and the reaction mixture was heated to 60 °C and stirred overnight. After cooling to ambient temperature, the reaction was quenched with aqueous glacial acidic acid (1 mL) and the solvent was removed under reduced pressure. The crude product was directly subjected to automated column chromatography (SiO_2_, CyH/aceton 50% then DCM/MeOH 0% to 10% linear gradient) to afford dye **12** (90.2 mg, 189 μmol, 59%) as a dark greenish solid.

**^1^H NMR** (600 MHz, DMSO-d6): δ = 8.18 (d, J = 7.8 Hz, 1H), 8.05 (s, br, 1H), 7.84 (t, J = 7.5 Hz, 1H), 7.76 (t, J = 7.7 Hz, 1H), 7.44 (d, J = 7.6 Hz, 1H), 6.94 – 6.89 (m, 2H), 6.87 (d, J = 9.0 Hz, 1H), 6.77 (d, J = 2.0 Hz, 1H), 5.78 (s, 1H), 3.69 – 3.60 (m, 4H), 3.40 – 3.31 (m, 2H), 3.26 (t, J = 5.3 Hz, 2H), 3.03 (q, J = 7.1 Hz, 2H), 1.23 – 1.17 (m, 3H), 1.21 (t, J = 7.1 Hz, 3H), 0.87 (t, J = 7.0 Hz, 3H) ppm.

**^13^C NMR** (151 MHz, DMSO-d6): δ = 154.4, 152.7, 151.9, 146.5, 134.0, 133.8, 132.5, 131.4, 130.6, 130.6, 130.3, 129.9, 128.2, 120.3, 120.1, 119.9, 115.1, 114.4, 102.4, 100.9, 94.6, 48.6, 47.3, 46.5, 46.3, 44.8, 43.7, 43.4, 18.8, 10.8, 8.7 ppm.

**HRMS** (ESI, pos.): *m/z* calculated for C_29_H_24_N_4_O_3_Na^+^ [M+Na^+^]: 499.1740, found 499.1761

**HRMS** (ESI, neg.): *m/z* calculated for C_29_H_23_N_4_O_3_^–^ [M-H^+^]^–^: 475.1775, found 475.1794.

Acid **12** (30.0 mg, 63.0 μmol, 1.0 equiv) was dissolved in DMF (2 mL) at ambient temperature. The mixture was treated with HATU (3.11 mg, 81.8 μmol, 1.30 equiv) and Et₃N (52.4 μL, 38.2 mg, 378 μmol, 6.0 equiv) and stirred for 15 min. Then, amine **5** (15.8 mg, 94.4 μmol, 1.50 eq.) was added and the resulting solution was stirred for overnight. The reaction was carefully quenched with saturated aqueous NaHCO_3_ (10 mL) and water (10 mL). The aqueous phase was extracted with DCM (3 x 50 mL), the combined organic phases were washed with brine (25 mL) and dried over anhydrous Na_2_SO_4_. The solvent was removed under reduced pressure and the crude product was purified by automated column chromatography (SiO2, CyH/acetone, 0% to 100%, linear gradient). The fractions containing the product were collected, the solvent was removed under reduced pressure and directly subjected to the subsequent saponification. The crude (35.0 mg) was dissolved in a mixture of THF/H_2_O (1:1, 11 mL) and NaOH (1M, 500 μL) was slowly added. The reaction mixture was stirred for 2 h and quenched by addition of glacial acidic acid (5 mL). The aqueous phase was extracted with DCM (2 x 20 mL), the combined organic phases were washed with brine (15 mL), dried over anhydrous Na_2_SO_4_ and the solvent was removed under reduced pressure. The crude product was purified RP-HPLC (H_2_O/MeCN 40% with 0.1% formic acid additive) to afford RhodaMal 638 **14** (22.0 mg, 38.2 μmol, 61% over two steps) as a dark green solid after lyophilization.

**^1^H NMR** (700 MHz, DMSO-d6): δ = 7.73 – 7.65 (m, 2H), 7.63 – 7.60 (m, 1H, major), 7.59 – 7.57 (m, 1H, minor), 7.49 – 7.46 (m, 1H, major), 7.45 – 7.42 (m, 1H, minor), 7.01 – 6.90 (m, 3H), 6.76 (s, 1H), 5.98 (s, 1H), 3.75 – 3.59 (m, 4H), 3.44 – 3.41; 3.38 – 3.35; 3.31 – 3.25; 3.20 – 3.03 (m, 6H, overlapped with residual water), 2.73 (s, 3H, major), 2.59 (s, 3H, minor), 2.02 – 1.97 (m, 2H, minor), 1.74 (t, J = 7.1 Hz, 2H, major), 1.64 – 1.53 (m, 2H, minor), 1.34 – 1.26 (m, 2H, major), 1.22 (t, J = 7.0 Hz, 3H), 0.92 (t, J = 7.0 Hz, 3H) ppm.

**^13^C NMR** (176 MHz, DMSO-d6, only major reported): δ = 172.7, 167.7, 154.3, 152.9, 152.0, 148.7, 146.81, 136.6, 133.8, 130.7, 130.1, 129.5, 129.1, 128.8, 127.2, 120.2, 120.1, 119.4, 115.1, 114.7, 103.6, 100.9, 94.6, 74.2, 63.0, 47.4, 46.6, 44.6, 43.2, 37.0, 31.0, 21.5, 10.8, 9.0 ppm.

**HRMS** (ESI, pos.): *m/z* calculated for C_34_H_33_N_5_O_4_Na^+^ [M+Na^+^]: 598.2425, found 598.2453.

**HRMS** (ESI, neg.): *m/z* calculated for C_34_H_23_N_5_O_4_^–^ [M-H^+^]^–^: 574.2460, found 574.2410.

RhodaMal 638 **14** (1.00 mg, 1.74 μmol, 1.0 equiv) was dissolved in DMF (200 μL). The mixture was treated with HATU (1m in DMF, 2.60 μL) and Et_3_N (1m in DMF, 13.9 μL, 8.0 equiv) subsequently and stirred for 5 min. Then, amine (0.25m in DMF, 13.9 μL, 2.0 equiv) was added and the reaction mixture was stirred overnight. The solvent was removed under reduced pressure and the crude product was directly subjected to RP-HPLC (H_2_O/MeCN 38%, with 0.1% formic acid additive), yielding RhodaMal 638-Mito (750 μg, 815 nmol, 47 %) as a dark greenish solid after lyophilization.

**HRMS** (ESI, pos.): m/z calc for C_58_H_69_N_6_O_3_P^+^ [M]^+^: 919.4459, found 919.4543.

# 5. Spectra

# 6. References

[1]	M. Tanioka, S. Kamino, N. Koga, D. Sawada, *J. Mater. Chem. C* **2020**, *8*, 543-549.

[2]	W. Chen, Q. Fang, D. Yang, H. Zhang, X. Song, J. Foley, *Anal. Chem.* **2015**, *87*, 609-616.

[3]	W. Chen, S. Xu, J. J. Day, D. Wang, M. Xian, *Angew. Chem. Int. Ed.* **2017**, *56*, 16611-16615.
